# Novel Type of Chronic Wasting Disease Detected in Moose (*Alces alces*), Norway

**DOI:** 10.3201/eid2412.180702

**Published:** 2018-12

**Authors:** Laura Pirisinu, Linh Tran, Barbara Chiappini, Ilaria Vanni, Michele A. Di Bari, Gabriele Vaccari, Turid Vikøren, Knut Ivar Madslien, Jørn Våge, Terry Spraker, Gordon Mitchell, Aru Balachandran, Thierry Baron, Cristina Casalone, Christer M. Rolandsen, Knut H. Røed, Umberto Agrimi, Romolo Nonno, Sylvie L. Benestad

**Affiliations:** Istituto Superiore di Sanità, Rome, Italy (L. Pirisinu, B. Chiappini, I. Vanni, M.A. Di Bari, G. Vaccari, U. Agrimi, R. Nonno);; Norwegian Veterinary Institute, Oslo, Norway (L. Tran, T. Vikøren, K.I. Madslien, J. Våge, S.L. Benestad);; Colorado State University, Fort Collins, Colorado, USA (T. Spraker);; Canadian Food Inspection Agency, Ottawa, Ontario, Canada (G. Mitchell, A. Balachandran);; Anses Lyon Unité “Maladies Neuro-Dégénératives,” Lyon, France (T. Baron);; Istituto Zooprofilattico Sperimentale del Piemonte Liguria e Valle d’Aosta, Torino, Italy (C. Casalone);; Norwegian Institute for Nature Research, Trondheim, Norway (C.M. Rolandsen);; Norwegian University of Life Sciences, Faculty of Veterinary Science, Oslo (K.H. Røed)

**Keywords:** prions, transmissible spongiform encephalopathy, chronic wasting disease, cervid, moose, reindeer, zoonoses, CWD, TSE, Alces alces, Norway, Nor16CWD

## Abstract

Chronic wasting disease (CWD) persists in cervid populations of North America and in 2016 was detected for the first time in Europe in a wild reindeer in Norway. We report the detection of CWD in 3 moose (*Alces alces*) in Norway, identified through a large scale surveillance program. The cases occurred in 13–14-year-old female moose, and we detected an abnormal form of prion protein (PrP^Sc^) in the brain but not in lymphoid tissues. Immunohistochemistry revealed that the moose shared the same neuropathologic phenotype, characterized by mostly intraneuronal deposition of PrP^Sc^. This pattern differed from that observed in reindeer and has not been previously reported in CWD-infected cervids. Moreover, Western blot revealed a PrP^Sc^ type distinguishable from previous CWD cases and from known ruminant prion diseases in Europe, with the possible exception of sheep CH1641. These findings suggest that these cases in moose represent a novel type of CWD.

Transmissible spongiform encephalopathies (TSEs), or prion diseases, are fatal and transmissible neurodegenerative diseases that include scrapie in sheep and goats, bovine spongiform encephalopathy (BSE) in cattle, chronic wasting disease (CWD) in cervids, and Creutzfeldt-Jakob disease in humans. TSEs are characterized by the misfolding of the normal host-encoded cellular prion protein (PrP^C^) into an abnormal disease-associated isoform (PrP^Sc^). PrP^Sc^ is considered to be the main or exclusive component of prions, the transmissible agents for TSEs (*1*). TSEs might have a genetic, infectious, or sporadic origin. Classical scrapie and CWD can be highly contagious, spreading directly among animals or through environmental contamination.

Since its first description in Colorado in 1967, CWD has been detected in new geographic areas and with increasing prevalence in captive and free-ranging cervids. Currently, the disease has been diagnosed in 25 states in the United States and in 2 Canada provinces (*2*,*3*), along with cases in South Korea associated with importation of infected cervids from Canada (*4*). High disease prevalence in some areas represents a challenge for preservation of wild cervids and mitigation of human exposure to CWD-related prions (PrP^Sc^). The high prevalence might be a plausible explanation for local deer population decline (*5*,*6*).

Species naturally affected by CWD include white-tailed deer (*Odocoileus virginianus*), mule deer (*O. hemionus*), moose (*Alces alces*), elk or wapiti (*Cervus canadensis*), and red deer (*C. elaphus*). In 2016, CWD was reported for the first time in Europe in wild reindeer (*Rangifer tarandus*) (*7*), a species never previously found to be naturally infected. The biochemical analysis and immunohistochemical (IHC) distribution of PrP^Sc^ from Norway reindeer revealed a pattern indistinguishable from North America isolates (*7*).

We report 3 cases of CWD detected in moose in Norway, characterized by biochemical and IHC features clearly different from CWD cases previously described in North America and Norway. Our findings suggest the involvement of a different type of CWD prion.

## Materials and Methods

### Animals and Tissues

The 3 moose were found in Trøndelag County in central Norway. The first case, moose no. 1 (ID P138), was emaciated and demonstrated abnormal behavior, showing reduced fear of humans. The second case, moose no. 2 (ID P153), was found dead in a river. Necropsy revealed normal body condition and pregnancy with twins; trauma was the cause of death. The third case, moose no. 3 (ID CD11399), was observed showing abnormal behaviors, including reduced fear of humans. Necropsy revealed a poor body condition and a severe dislocation of the left hip joint, which might have influenced the animal’s behavior. All 3 were older female moose (13, 14, and 13 years old, based on counts of cementum annuli in the root of the first incisor [*8*]).

Samples included in this study are described in the Table. We performed the primary diagnostic test (TeSeE ELISA; Bio-Rad Laboratories, Inc., Hercules, CA, USA) for detection of protease-resistant core of PrP^Sc^ (PrP^res^) on the 3 moose and 1 reindeer from Norway (*7*) in the medulla oblongata at the level of the obex. After the initial positive test results, the remaining brain tissues were divided; one half was fixed in 10% neutral buffered formalin, and the other half was frozen. In addition, lymph nodes (Ln) from moose no. 1 (retropharyngeal, submandibular, and jejunal Ln), moose no. 3 (retropharyngeal, parotid, prescapular, and submandibular Ln, and tonsils), and the reindeer (2 tracheobronchial Ln) were equally divided and formalin fixed or frozen.

**Table:** Summary characteristics of samples used in a study characterizing chronic wasting disease in moose (*Alces alces*), Norway*

Animal or species	Geographic origin	Age	Pathologic phenotype	PrP genotype†
Moose no. 1	Norway	13 y	Atypical CWD	KK_109_MM_209_
Moose no. 2	Norway	14 y	Atypical CWD	KK_109_MM_209_
Moose no. 3	Norway	13 y	Atypical CWD	KK_109_MM_209_
Reindeer	Norway	3–4 y	CWD	VV_2_GG_129_SS_138_VV_169_
Elk or wapiti	Canada	6 y	CWD	MM_132_
Elk or wapiti	Canada	Adult	CWD	MM_132_
Elk or wapiti	Canada	6 y	CWD	MM_132_
White-tailed deer	Canada	4 y	CWD	GG_96_
Moose	Canada	Adult	CWD	KK_109_II_209_‡
Sheep	Italy	2.5 y	Classical scrapie	AA_136_RR_154_QQ_171_
Sheep	Italy	Adult	Classical scrapie	AA_136_RR_154_QQ_171_
Sheep	Italy	4.5 y	Atypical/Nor98 scrapie	AA_136_RH_154_QQ_171_
Sheep	Experimental sample	NA	Scrapie CH1641§	AA_136_HH_154_QQ_171_
Sheep	France	NA	Scrapie CH1641-like¶	AA_136_RR_154_QQ_171_
Cattle	Italy	6 y	C-BSE	NA
Cattle	Italy	15 y	L-BSE	NA
Cattle	France	11 y	H-BSE	NA

*All samples were from naturally infected animals, with the exception of 1 sheep experimentally inoculated with CH1641. BSE, bovine spongiform encephalopathy; C-BSE, classical BSE; CWD, chronic wasting disease; H-BSE, H-type atypical BSE; L-BSE, L-type atypical BSE; NA, not available; PrP, prion protein. †The PrP genotype reported is expressing the amino acid variants present at the major polymorphic codons described for each species. ‡The Canada isolate was homozygous for lysine at codon 109 and for isoleucine at codon 209 (KK_109_II_209_) (GenBank accession no. MH230114). §Reference (*9*). ¶Reference (*10*).

### Genotyping of Moose *PRNP*

DNA was extracted from 100 mg of brain tissue by using a DNeasy Blood and Tissue Kit (QIAGEN, Hilden, Germany), according to the manufacturer’s instructions. The *PRNP* coding sequence was amplified in a 50 µL final volume by using 5 µL of extracted DNA, eluate 1X AmpliTaq Gold 360 PCR Buffer (Life Technologies, Carlsbad, CA, USA), 2.5 mmol/L MgCl_2_, 1X 360 GC Enhancer (Life Technologies), 200 µmol/L dNTPs, 0.25 µmol/L of forward (5′-GCTGACACCCTCTTTATTTTGCAG-3′) and reverse (5′-GATTAAGAAGATAATGAAAACAGGAAG −3′) primers (*11*), and 0.5 µL AmpliTaq Gold 360 (Life Technologies), according to the following amplification protocol: 5 min at 96°C; 30 s at 96°C; 15 s at 57°C; 90 s at 72°C for 40 cycles; and 4 min at 72°C. Amplicons were purified with the Illustra ExoProStar 1-Step clean-up kit (GE Healthcare Life Sciences, Little Chalfont, UK), sequenced using the Big Dye Terminator Cycle Sequencing Kit v1.1 (Life Technologies), and purified with the Big Dye XTerminator Purification Kit (Life Technologies), and detected by using an ABI PRISM 3130 apparatus (Life Technologies). 

### Anti–Prion Protein Monoclonal Antibodies

Several antibodies with different epitopes (sheep prion protein [PrP] numbering) were used for discriminatory Western blot (WB) and IHC. SAF84 (aa 167–173) was obtained from Bertin Pharma (Montigny-le-Bretonneux, France), L42 (aa 148–153) from R-Biopharm (Darmstadt, Germany), 9A2 (aa 102–104) and 12B2 (aa 93–97) from Wageningen Bioveterinary Research (Lelystad, Netherlands), and F99/97.6 (aa 220–225) from VMRD, Inc. (Pullman, WA, USA).

### Immunohistochemistry

Brain, Ln, and tonsil tissues were formalin fixed for >48 h and processed by standard histopathologic techniques. We used IHC to visualize the distribution of PrP^Sc^ as previously described (*7*). We applied a commercially available kit (EnVisionTM + System HRP [AEC]; DAKO, Glostrup, Denmark) by using the monoclonal antibodies (mAbs) 12B2, 9A2, L42, SAF 84, or F99/97.6 for 30 min at 37°C and counterstained with hematoxylin. In each run, tissue from CWD-negative moose and reindeer were added as negative controls.

### PrP^res^ Detection 

We initially tested the obex samples by using the TeSeE SAP ELISA and confirmed positive ELISA results by using the TeSeE WB. Both tests were performed as recommended by the manufacturer (Bio-Rad).

### Typing of PrP^res^

We performed further characterization of PrP^res^ by discriminatory immunoblotting, according to the ISS discriminatory WB method (*12*). Brain homogenates at 10% (wt/vol) in 100 mmol/L Tris-HCl (pH 7.4) 2% sarkosyl were incubated for 1 h at 37°C with Proteinase K (Sigma-Aldrich, St. Louis, Missouri, USA) to a final concentration of 200 µg/mL. Protease treatment was stopped with 3 mmol/L PMSF (Sigma-Aldrich). Aliquots of samples were added with an equal volume of isopropanol/butanol (1:1 vol/vol) and centrifuged at 20,000 × *g* for 10 min. The pellets were resuspended in denaturing sample buffer (NuPAGE LDS Sample Buffer; Life Technologies) and heated for 10 min at 90°C.

We loaded each sample onto 12% bis-Tris polyacrylamide gels (Invitrogen) for electrophoresis with subsequent WB on polyvinylidene fluoride membranes using the Trans-Blot Turbo Transfer System (Bio-Rad) according to the manufacturer’s instructions. The blots were processed with anti-PrP mAbs by using the SNAP i.d. 2.0 system (Millipore, Burlington, MA, USA) according to the manufacturer’s instructions. After incubation with horseradish peroxidase–conjugated anti–mouse immunoglobulin (Pierce Biotechnology, Waltham, MA, USA) at 1:20,000, the PrP bands were detected by using enhanced chemiluminescent substrate (SuperSignal Femto; Pierce Biotechnology) and ChemiDoc imaging system (Bio-Rad). The chemiluminescence signal was quantified by using Image Lab 5.2.1 (Bio-Rad).

We performed deglycosylation by adding 18 μL of 0.2 mmol/L sodium phosphate buffer (pH 7.4) containing 0.8% Nonidet P40 (Roche) and 2 μL (80 U/ml) di N-Glycosidase F (Roche) to 5 μL of proteinase K–digested and denaturated samples. We then incubated the mixtures for 3 h at 37°C with gentle shaking.

## Results

CWD was diagnosed in 2 moose in May 2016 in Norway’s Selbu municipality and in 1 moose in October 2017 in Lierne municipality. Selbu and Lierne are respectively located ≈300 and ≈450 km northeast of Nordfjella, where CWD in reindeer was detected in 2016. Norway is populated by several species of wild cervids with varying degrees of overlapping range. Seasonal migrations are common and distances might exceed 150 km (*13*–*15*). However, studies tracking global positioning satellite–collared moose have not documented regular seasonal migrations between Selbu and Lierne municipalities, suggesting that these can be considered different moose subpopulations.

We initially detected PrP^Sc^ in brain samples by using a rapid test and then confirmed by WB (data not shown) and IHC. Sequencing analysis of the entire PrP coding sequence revealed that the 3 moose had the wild type PrP genotype, homozygous for lysine at codon 109 and for methionine at codon 209 (KK_109_MM_209_) (GenBank accession no. MH230115).

### Discriminatory PrP^Sc^ Immunohistochemistry Show Differences between Reindeer and Moose

**Figure 1:**
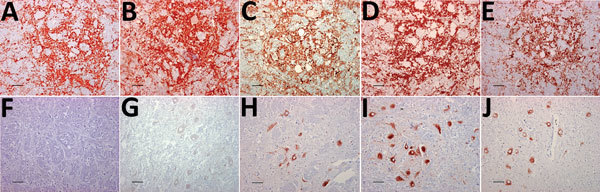
Immunohistochemical detection of disease-associated prion protein in brain sections at the level of the obex in cervids with chronic wasting disease, Norway. A–E) Reindeer; F–J) moose. mAbs used were 12B2 (A, F), 9A2 (B, G), L42 (C, H), SAF 84 (D, I), and F99/97.6 (E, J). Staining obtained in the reindeer tissues is similar regardless of mAbs used (A–E). Conversely, for moose tissues, the staining was primarily observed intraneuronally with L42, SAF84, and F99/97.6 (H–J) but was not observed using the more N-terminal mAbs 12B2 and 9A2 (F, G). Scale bars indicate 40 µm. mAbs, monoclonal antibodies.

The distribution of PrP^Sc^ staining was examined by IHC and compared in the tissues of the 3 moose and the reindeer by using 5 different antibodies (Figure 1). No staining was observed in CWD-negative reindeer and moose independently of the antibody used. The distribution of PrP^Sc^ in the reindeer was identical for each of the 5 antibodies and did not differ from the description of PrP^Sc^ distribution in North America cervids (*16*–*18*). The labeling was most consistent within the gray matter of the medulla oblongata, particularly in the dorsal motor of the vagus nerve (*7*). The thalamic and brain stem regions of the brain were most affected, with a minimal amount of PrP^Sc^ identified dorsal to the corpus callosum.

PrP^Sc^ labeling in the moose brains (Figure 1, panels F–J) was clearly different from that of the reindeer (Figure 1, panels A–E). In the moose, after staining with F99/97.6 and L42, PrP^Sc^ was almost exclusively observed as intraneuronal aggregates, although intraastrocytic type (multiple small granules scattered in the cytoplasm of astrocyte-resembling cells) and intramicroglial type (1 single or a few large granules in close proximity to microglia-like nuclei) were also observed in the cerebral cortices and olfactory bulb (Technical Appendix Figure 1). The degree of PrP^Sc^ staining was more intensive and appeared more widespread in the neuropil using SAF84.

At the level of the obex, we found stained neurons in all nuclei, whereas the dorsal motor of the vagus nerve was not remarkably stained, as observed in reindeer. The intensity of labeling varied among the 3 moose; no. 2 displayed sparse labeling, no. 3 widespread and abundant labeling, and no. 1 intermediate labeling intensity. We observed PrP^Sc^ in all parts of the brain investigated except the cerebella of moose nos. 1 and 2. A diffuse or discrete punctate staining was observed in the granular layer of the cerebellum of moose no. 3, with stronger staining in some Golgi neurons (Technical Appendix Figure 1, panel B). In all 3 moose, the cortical regions showed laminar staining of neurons in all the cell layers, especially in fusiform-shaped neurons. The neurons of the olfactory tubercle from all 3 also stained strongly, and some glia-associated staining could be observed.

In contrast to the reindeer, the downstream flexible tail mAbs 12B2 and 9A2 did not stain in the moose (Figure 1, panels F and G), suggesting that the moose PrP^Sc^ was truncated by endogenous proteases further upstream in the N terminus than was reindeer PrP^Sc^. Contrary to previous findings in reindeer, PrP^Sc^ was not detected in the Ln from moose no. 1 or in the Ln and tonsils from moose no. 3 (lymphoid tissues were not available in moose no. 2) by either IHC or ELISA.

### PrP^Sc^ from Norway Moose Compared with Other CWD Isolates from Canada and Norway

**Figure 2:**
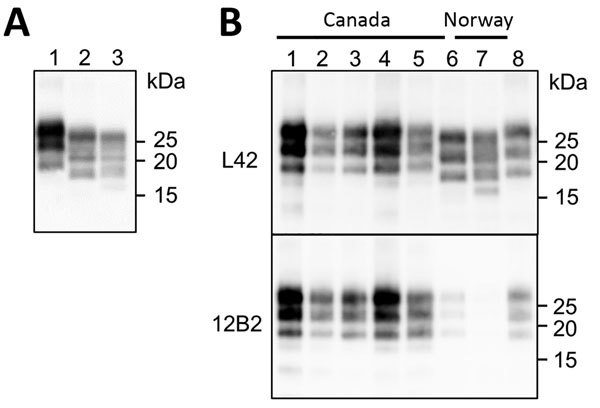
Western blot analysis of PrP^res^ in brains of chronic wasting disease–affected cervids from Norway and Canada. A) Western blot analysis PrP^res^ in brains of moose and reindeer from Norway. Membrane was probed with L42 monoclonal antibodies. Molecular weights (kDa) are indicated on the right. Tissue equivalent loaded per lane was 1 mg. B) Western blot analysis of PrP^res^ from moose isolates from Norway (lanes 6–7) compared with PrP^res^ from chronic wasting disease–affected elk or wapiti (lanes 1–3), white-tailed deer (lane 4), and moose (lane 5) from Canada. Membranes were probed with L42 (upper) and 12B2 (lower) monoclonal antibodies. A scrapie sheep sample from Italy was added as control (lane 8). Molecular weights (kDa) are indicated on the right of each blot. Tissue equivalents loaded per lane were 1 mg for Canadian isolates, 2 mg for Norwegian isolates, and 0.15 mg for scrapie sheep control. PrP^res^, protease-resistant core of abnormal form of prion protein.

We compared the PrP^Sc^ features in moose from Norway with those of other CWD isolates from Norway and Canada by discriminatory WB, which enabled comparison of PrP^res^ by epitope mapping with different antibodies. Norway moose PrP^res^ had a lower apparent molecular weight (MW) than PrP^res^ from Norway reindeer (Figure 2, panel A) or from Canada isolates (Figure 2, panel B). This lower MW was explained by the occurrence of more C-terminal cleavage of PrP^Sc^ by protease K, as confirmed by the partial loss of the 12B2 epitope (Figure 2, panel B).

**Figure 3:**
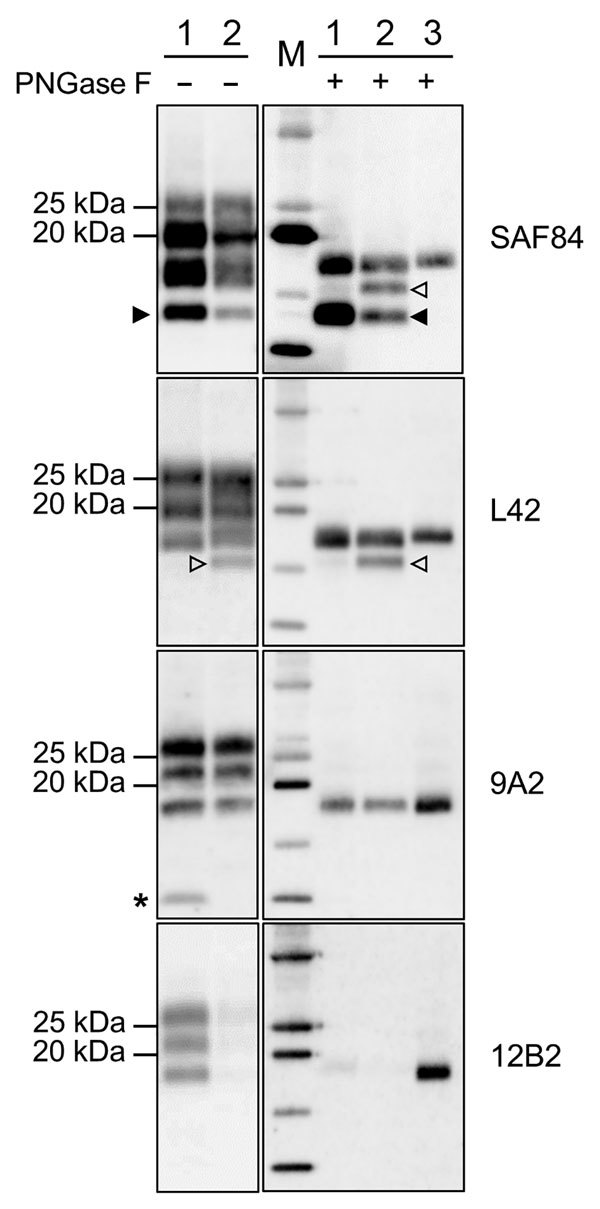
Characterization of PrP^res^ fragments from moose (*Alces alces*) in Europe by epitope mapping. Mapping with mAbs spanning the whole prion protein enabled the analysis of PrP^res^ in moose samples before (PNGase F–) and after (PNGase F+) deglycosylation, based on presence or absence of the epitopes and apparent molecular weight. Lanes 1, moose no. 1; lanes 2, moose no. 3; lane M, protein standards; lane 3, sheep scrapie sample. Solid arrowheads indicate C-terminal fragment of ≈13 kDa fragment (present in both samples and detected with SAF84 mAbs). Open arrowheads indicate C-terminal fragment of ≈16 kDa fragment in moose no. 2 with SAF84 and L42 mAbs. Asterisk indicates the internal fragment detected in moose no. 1 with 9A2 mAbs. Molecular weights are indicated on the left. In the blots on the right, protein standards are shown in lane M (10, 15, 20, 25, and 37 kDa). The mAbs used are indicated on the right. mAbs, monoclonal antibodies; PrP^res^, protease-resistant core of abnormal form of prion protein.

Given the unusual pattern observed in moose isolates from Norway, we further investigated their biochemical characteristics with additional mAbs and by enzymatic deglycosylation (Figure 3). Moose samples showed a main C-terminal fragment of ≈17 kDa, detected with SAF84, L42, and 9A2, and an additional glycosylated C-terminal fragment of ≈13 kDa (CTF13) detected only with SAF84. The N terminal 12B2 epitope was mainly lost, although a small amount of PrP^res^ was still detectable in moose no. 1 (Figure 3) and no. 3 (Technical Appendix Figure 2) with this antibody.

In moose nos. 2 and 3, an additional glycosylated C-terminal fragment of ≈16 kDa (CTF16) was detected by SAF84 and L42 mAbs (Figure 3; Technical Appendix Figure 2). We cannot exclude that a small amount of CTF16 was also present in moose no. 1, given that a weak PrP^res^ fragment of ≈16 kDa was detectable upon deglycosylation and long exposure of blots (Figure 3; Technical Appendix Figure 3). Moose nos. 1 and 3 also had a nonglycosylated internal fragment of ≈10 kDa, cleaved at both N and C termini of PrP^Sc^, which was recognized by using mAbs 9A2 (Figure 3). Moreover, the analysis of PrP^res^ from different neuroanatomic regions showed that the slight differences observed among the 3 moose were not dependent on the area analyzed (Technical Appendix Figure 2).

### Comparison of the PrP^Sc^ Features of the Norway Moose with Sheep and Cattle Prion Strains from Europe

**Figure 4:**
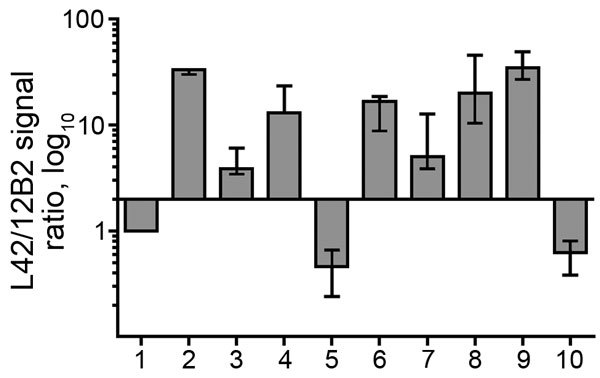
Bar graph of antibody-signal ratios (y-axis) showing discrimination of the ovine, bovine, moose, and reindeer samples (x-axis) analyzed in a study characterizing chronic wasting disease in moose (*Alces alces*), Norway. Numbers indicate sample type: 1, scrapie; 2, CH1641; 3, CH1641-like; 4, classical bovine spongiform encephalopathy (BSE); 5, H-type atypical BSE; 6, L-type atypical BSE; 7, moose no. 1; 8, moose no. 2; 9, moose no. 3; 10, reindeer. The antibody ratio is the L42/12B2 ratio of the chemiluminescence signal relative to the L42/12B2 ratio of the control scrapie loaded in each blot. Bars represent median values of >3 independent determinations; error bars represent the range of observed values. Bars start at y = 2, which is the cutoff value of the antibody ratio for the discrimination of low molecular weight samples (i.e., suspected bovine spongiform encephalopathy cases) from scrapie, according to discriminatory Western blot.

**Figure 5:**
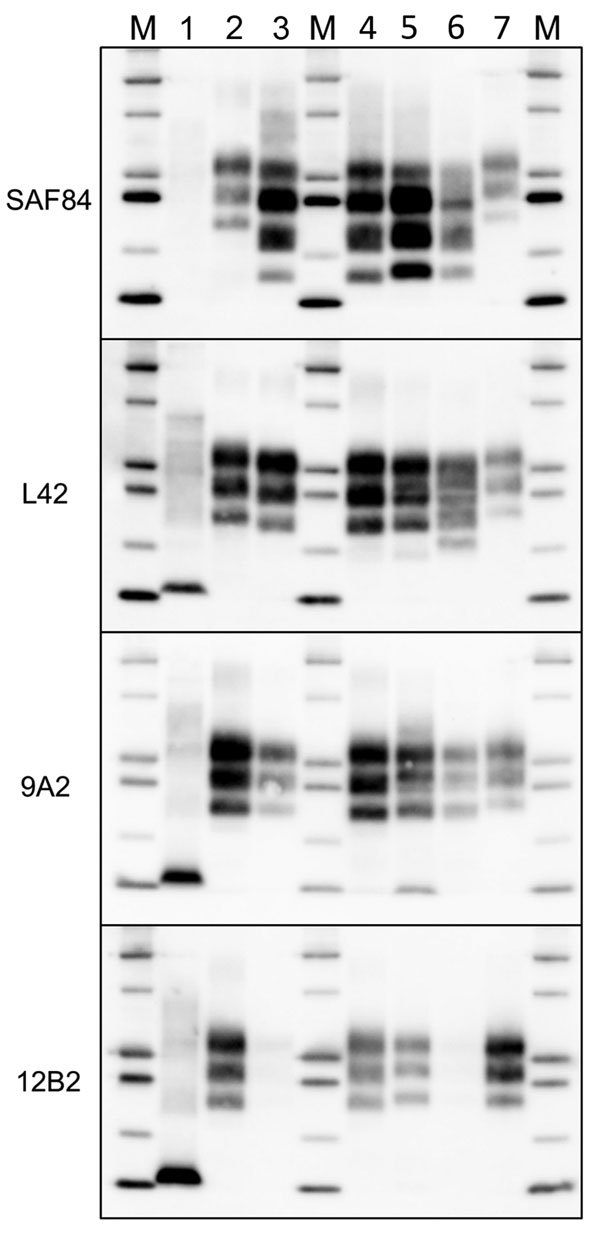
Comparison of protease-resistant PrP^res^ from moose (*Alces alces*) with chronic wasting disease and from sheep with scrapie, Europe. Representative blots show epitope mapping analysis of PrP^res^ (lane 4, CH1641; lane 5, moose no. 1; lane 6, moose no. 2) in comparison with different ovine transmissible spongiform encephalopathy isolates (lane 1, atypical/Nor98; lane 2, classical scrapie; and lane 3, CH1641). A chronic wasting disease isolate from Canada was loaded as control (lane 7). The antibodies used are indicated on the left. Protein standards are shown in lane M (10, 15, 20, 25, 37, and 50 kDa). The small amount of PrP^res^ with intact 12B2 epitope in moose no.1 had a molecular weight higher than that observed with more C-terminal monoclonal antibodies (18.7 +0.3 kDa measured with 12B2 vs. 17.2 +0.1 kDa measured with L42). Even if the increase of the apparent molecular weight might be a known behavior when proteinase K cleavage occurs near the epitope, we noted that, in the case of moose no. 1, the 12B2-positive PrP^res^ had a molecular weight higher than scrapie (18.1 +0.1 kDa measured with 12B2) and CH1641-like sample (18.1 +0.4 kDa when detected with 12B2). PrP^res^, protease-resistant core of abnormal form of prion protein.

Comparison with ovine and bovine prions was performed to determine the N terminal cleavage of the main PrP^res^ fragment by analyzing the different PrP^res^ fragments in each sample, the MW of these fragments, and the L42/12B2 antibody ratio (Figure 4; Technical Appendix Table). Among ovine prions, classical scrapie and atypical/Nor98 were easily discriminated from moose isolates (Figure 5). Classical scrapie PrP^res^ had a higher MW than moose PrP^res^, as confirmed by the preservation of 12B2 epitope. As previously observed (*19*), Nor98 PrP^res^ was cleaved at both the N and C termini, and the characteristic 11–12 kDa band was detected by L42, 9A2, and 12B2 mAbs (Figure 5). In contrast, CH1641 samples showed molecular features partially overlapping with the moose (Figure 5). CH1641 samples showed a PrP^res^ of ≈17 kDa and were accompanied by an additional C-terminal fragment of 13–14 kDa detected by using SAF84 mAbs (*20*). However, CTF16 and the internal PrP^res^ fragment of 10 kDa could not be detected in CH1641 samples.

**Figure 6:**
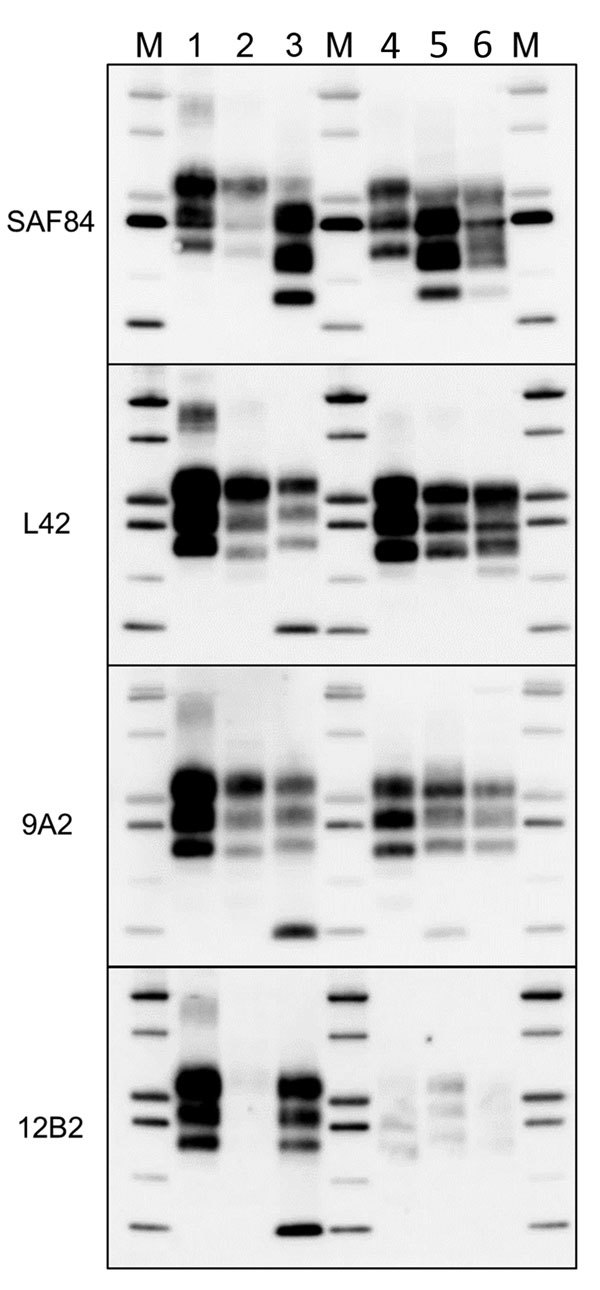
Comparison of protease-resistant core of abnormal form of prion protein from moose (*Alces alces*) in Europe with chronic wasting disease and from cattle with BSE. Representative blots show epitope mapping analysis of protease-resistant core of abnormal form of prion protein in moose (lane 5, moose no. 1; lane 6, moose no. 2) in comparison with different BSE isolates (lane 2, classical BSE; lane 3, H-type BSE; and lane 4, L-type BSE). A sheep scrapie isolate was loaded as control (lane 1). The antibodies are indicated on the left. Protein standards are shown in lane M (10, 15, 20, 25, 37, and 50 kDa). BSE, bovine spongiform encephalopathy.

Moose PrP^Sc^ did not overlap with any type of bovine PrP^Sc^. The lack of the 12B2 epitope in moose PrP^res^ was similar to C-type and atypical L-type BSE, but the 2 bovine prions had neither CTF13, CTF16, nor the internal fragment (Figure 6). H-type atypical BSE showed the CTF13 and the internal fragment similar to moose PrP^res^, but the main PrP^res^ fragment showed a higher MW and preserved the 12B2 epitope (Figure 6).

The ratio of reactivity obtained with L42 and 12B2 antibodies reflected the N terminal cleavage of the main fragment of PrP^Sc^, enabling confirmation that the differences observed in MW of PrP^res^ actually depend on different N terminal proteinase K cleavages, irrespective of the host species (Figure 4). Values >2 are indicative of BSE-like cleavage, whereas values <1 indicate a better preservation of 12B2 epitope compared with scrapie. In this respect, the behavior of moose PrP^res^ was BSE-like (ratio >2). However, moose no. 1 had a ratio lower than moose nos. 2 and 3. The CH1641-like field sample was similar to moose no. 1 in this respect, whereas CH1641 was similar to moose nos. 2 and 3. Finally, the value <1 observed for PrP^res^ in H-type atypical BSE, CWD in reindeer, and CWD isolates from Canada reflected their higher MW compared with classical scrapie (online Technical Appendix Table).

## Discussion

Although CWD has been detected in several captive and free-ranging cervid species from a large geographic area in North America, <10 cases in moose have been reported (*21*–*23*). We report 3 naturally occurring cases of prion disease in moose in Norway that showed molecular and IHC phenotypes differing from those previously described for classical CWD in North America, as well as in reindeer in Norway. The phenomenon of strain variation is well known in prion diseases and is often associated with phenotype variation in natural hosts, as observed in bovines with classical, H-type, or L-type BSE, and in sheep with classical or atypical/Nor98 scrapie. Identification of a new CWD phenotype in 3 moose in Norway can be suggestive of a new CWD strain. Although the existence of CWD strain variation in North America has been inferred from transmission studies (*24*–*26*), this phenomenon has not been directly associated with phenotypic variations in natural hosts so far.

The phenotype variant found in moose from Norway could be hypothetically attributed to host species factors. To address this issue, we directly compared PrP^Sc^ characteristics in the Norway moose with those in a Canada moose with CWD. In agreement with the available evidence, we found that the Canada moose PrP^Sc^ had features different from Norway moose PrP^Sc^ and were indistinguishable from other cervids with classical CWD. This finding suggests that the variant PrP^Sc^ type observed in Norway moose could not simply reflect a host species factor. Notably, in both natural and experimental conditions, CWD-affected moose in North America have been reported to display disease features indistinguishable from CWD in other cervids and had detectable PrP^Sc^ in lymphoid tissues (*21*,*27*).

Species-specific amino acid polymorphisms in the cervid PrP are associated with CWD susceptibility, incubation time, and pathology (*28*–*30*). In transmission experiments, atypical features were reported in elk or wapiti and mule deer with genotypes associated with a relative resistance to disease, extension of the incubation period, or both (*31*,*32*). Moose PrP is polymorphic at codon 109 (K/Q) and 209 (M/I), combined in 3 alleles: K_109_M_209_ (observed in Europe and North America), Q_109_M_209_ (observed in Europe), and K_109_I_209_ (observed in North America) (*33*,*34*). The 3 moose with CWD from Norway had the KK_109_MM_209_ genotype, whereas the moose case from Canada used for comparison had the KK_109_II_209_ genotype. Thus, we cannot exclude that the differences observed between Norway and Canada moose in our study are dependent on differences in PrP genotype. However, a classical CWD phenotype has been reported in naturally (*21*) and experimentally infected (*27*) moose with the KK_109_MM_209_ genotype, suggesting that a difference at PrP codon 209 is probably not the cause of the variant phenotype observed in moose in Norway. All of these findings suggest that neither the species nor the individual PrP genotypes are likely to have caused the variant phenotypes observed and imply that this variant phenotype could represent a novel CWD strain.

CWD is known to be a highly contagious disease in North America; however, data relating to the disease in moose are sparse and insufficient to understand the epidemiology and the implications of CWD in this species. The apparent low CWD prevalence reported for moose in North America compared with other cervid species might be attributable to the individual social behavior of moose and the minimal habitat overlap between moose and other cervids in areas with CWD. Additionally, surveillance program design, disease variability, and host genetics might influence the prevalence of the disease. Based on the epizootic dynamics in North America, CWD plausibly could have become established in reindeer in Norway more than a decade ago (*35*). In this scenario, the disease in moose could possibly be linked to the disease observed in reindeer, with strain mutation or phenotype shift putatively caused by interspecific transmission. However, a main cause of strain mutation after interspecies transmission (i.e., PrP amino acid differences between the donor and host species) is not relevant in this case because reindeer and moose share the same PrP primary sequence. An alternative hypothesis could be that moose have a prion disease that is independent of the reindeer epidemic, being either specific to the Norwegian moose or acquired by species other than the reindeer.

The 3 moose were 13, 14, and 13 years of age. Although moose can reach ages beyond 20 years, we consider these moose as old because female moose >10–12 years of age start to show signs of senescence and declining survival and reproduction rates (*36*,*37*). The old age of the moose, the absence of lymphoid tissue involvement, and the low disease prevalence observed so far (3 of 10,531 moose tested) could suggest that CWD in moose is less contagious than classical CWD or could represent a spontaneous TSE. The finding that the affected moose were from the same geographic area does not seem to support a spontaneous origin of the disease; however, the actual evidence for geographic clustering could have been biased by oversampling in Trøndelag County, where the first positive moose was detected. Lack of detailed data on the ages of the moose tested so far in different geographic areas prevents any definitive conclusion. Still, the recent detection of a positive moose in Finland, several hundred kilometers from Trøndelag County, might indicate that the disease is not restricted to Norway (*38*). The ongoing intensive surveillance in Norway and several European Union countries with large moose populations will help to better clarify the actual geographic distribution and prevalence and will be critical for understanding the contagious or spontaneous nature of the disease.

The 3 moose analyzed shared a distinctive IHC pattern, mainly characterized by intraneuronal accumulation of PrP^Sc^, and common PrP^Sc^ features, such as the proteinase K N-terminal cleavage and the presence of an additional CTF13 fragment. However, we also observed unexpected differences among the 3 moose. By WB, the CTF16 fragment was observed in moose nos. 2 and 3 but not in moose no. 1, whereas the nonglycosylated internal fragment of 10 kDa was evident in moose nos. 1 and 3 but could not be detected in moose no. 2. Furthermore, we also showed that these differences did not depend on the brain area investigated. We cannot rule out that these slight differences might depend on technical issues rather than represent actual PrP^Sc^ variations. The outcome of the ongoing bioassay experiments will help to clarify the meaning of the observed variations.

By comparing the moose PrP^Sc^ features with other animal TSEs circulating in Europe, we found no evidence of similarities with bovine and ovine prions. Minimal similarities were observed with CH1641 samples; however, CH1641 cases have not yet been detected in Norway. Bioassay in a large spectrum of rodent models will assist in determining whether these molecular similarities imply biologic association between the atypical CWD in moose and small ruminant CH1641. Transmission studies in several rodent models are under way and will help to clarify whether the different phenotype observed (designated Nor16CWD) could reflect the presence of a new cervid prion strain in moose from Norway.

Technical Appendix Molecular weight of protease-resistant core of abnormal form of prion protein (PrP^res^) fragments determined by epitope mapping, immunohistochemistry staining of moose brain tissues of a Norwegian moose with chronic wasting disease, Western blot analysis of PrP^res^ from different brain areas brain of 3 Norwegian moose with chronic wasting disease, and Western blot analysis of PrP^res^ fragments after deglycosylation from a Norwegian moose brain.
