# Supplementary material for: Novel Type of Chronic Wasting Disease Detected in Moose (Alces alces), Norway
Source: Emerg Infect Dis. 2018 Dec;24(12):2210–8. doi: 10.3201/eid2412.180702 (PMC6256397; doi:10.3201/eid2412.180702)
Supplement: Technical Appendix — Molecular weight of protease-resistant core of abnormal form of prion protein (PrPres) fragments determined by epitope mapping, immunohistochemistry staining of moose brain tissues of a Norwegian moose with chronic wasting disease, Western blot analysis of PrPres from different brain areas brain of 3 Norwegian moose with chronic wasting disease, and Western blot analysis of PrPres fragments after deglycosylation from a Norwegian moose brain. [file 18-0702-Techapp-s1.pdf]

Article DOI: https://doi.org/10.3201/eid2412.180702

Novel Type of Chronic Wasting Disease Detected in Moose (*Alces alces*), Norway

Technical Appendix

Technical Appendix Table. Molecular weight of PrP^{res} fragments*

Sample	Main fragment (L42)	CTF13 (SAF84)	CTF16 (L42)	Internal fragment† (9A2)
Scrapie	17.8 ±0.1			
Reindeer	18.9 ±0.4			
Moose #1	17.2 ±0.1	13.0 ±0.1		10.0 ±0.0
Moose #2	17.1 ±0.2‡	12.9 ±0.1	15.6 ±0.1	
Moose #3	17.3 ±0.1‡	13.1 ±0.2	15.7 ±0.2	10.2 ±0.1
CH1641 (exp)	17.3 ±0.2	12.4 ±0.0		
CH1641-like	17.1 ±0.1	12.5 ±0.0		
Atypical scrapie/Nor98				10.8 ±0.2
C-BSE	17.2 ±0.0			
L-BSE	17.3 ±0.1			
H-BSE	18.2 ±0.0	12.6 ±0.2		10.0 ±0.0

*The molecular weight (MW), indicated in kDa, of all PrP^{res} fragments was determined by epitope mapping with three mAbs (SAF84, L42, 9A2), before and after deglycosylation. For each type of fragment, the best mAb was chosen to avoid epitopes near the cleavage site of PK. The MW was measured by comparison with molecular weight standard run in three lanes of each gel and it is expressed as mean (at least from three different experiments)±standard deviation.
†The internal fragment refers to the PrP^{Sc} fragment resulting after C- and N terminal cleavage by protease K.
‡The MW of the main fragment in moose #2 and #3 was not possible to determine with L42 because of the presence of the glycosylated forms of CTF16. For these samples the molecular weight of the main fragment was determined after deglycosylation.

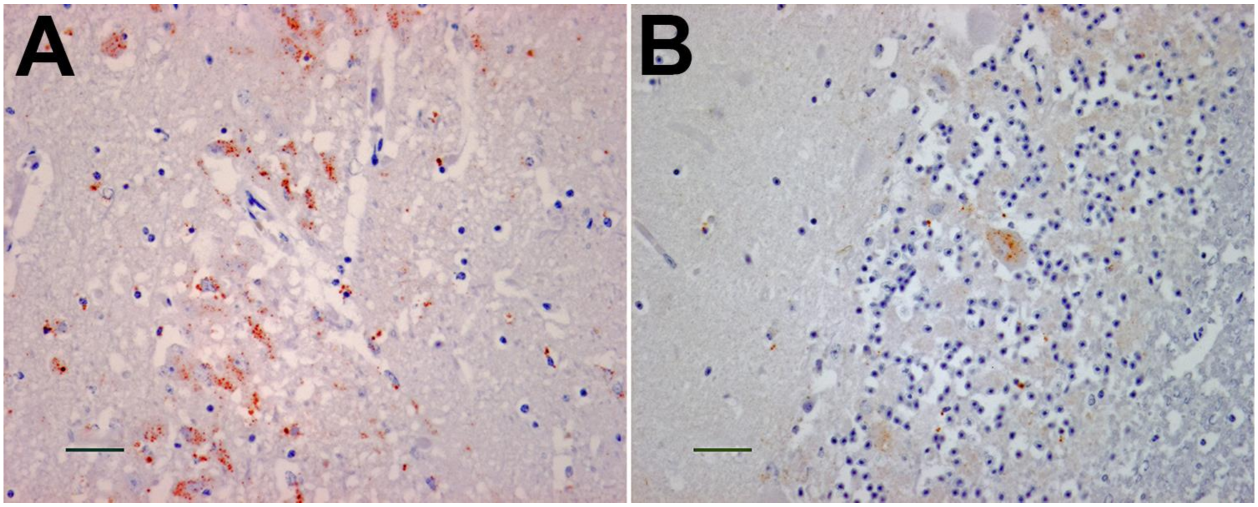

Technical Appendix Figure 1. IHC staining of moose brain tissues using mAb L42: A, intraneuronal and glia-associated staining in the olfactory bulb. B: staining in a Golgi cell in the granular layer of the cerebellum. Bars: 20 µm.

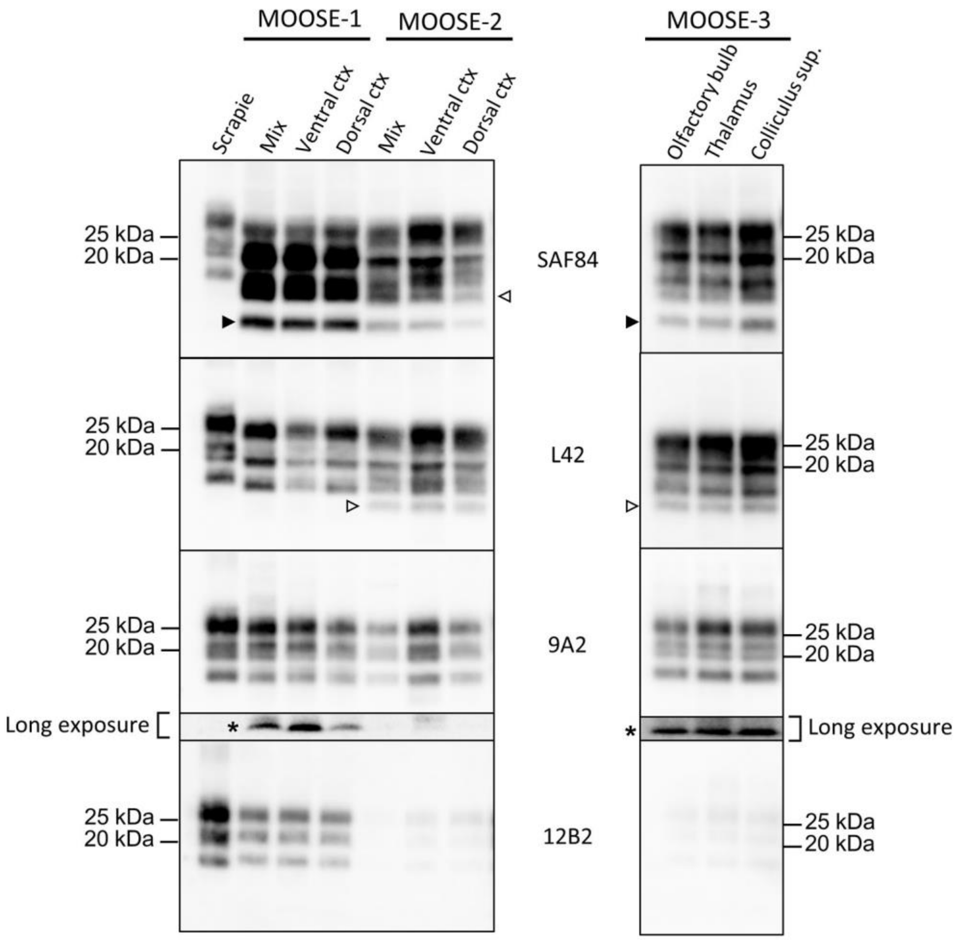

Technical Appendix Figure 2. Western blot analysis of PrP^{res} from brain of three moose. Different brain areas were analyzed with four mAbs spanning the PrP protein (SAF84, L42, 9A2, 12B2). Filled arrows indicate CTF13 fragment, empty arrows indicate CTF16 fragment in moose #2 and #3 with SAF84 and L42, asterisk indicate the internal fragment detected in moose #1 and #3 with 9A2. The lower panel in 9A2 blot shows the results obtained around 10 kDa at a longer exposure.

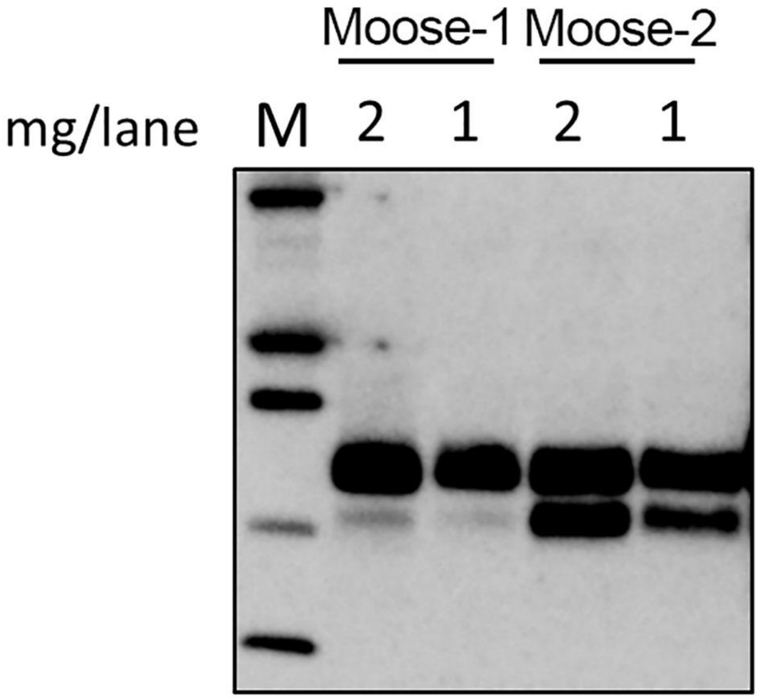

Technical Appendix Figure 3. Western blot analysis of PrP^{res} fragment from moose #1 and moose #2 after deglycosylation. Tissue equivalents loaded per lane were 2 mg (indicated as "2") and 1 mg after dilution ("1"). Protein standards are indicated as "M" (10, 15, 20, 25, 37 kDa). Membrane was probed with L42.
